# Supplementary material for: Endothelial Biomarkers Are Superior to Classic Inflammatory Biomarkers in Community-Acquired Pneumonia
Source: Biomedicines. 2024 Oct 21;12(10):2413. doi: 10.3390/biomedicines12102413 (PMC11505377; doi:10.3390/biomedicines12102413)
Supplement: Supplementary file 1 [file biomedicines-12-02413-s001.zip › biomedicines-3268672-supplementary.pdf]

SUPPLEMENTAL DATA

Supplemental Data Table S1. Values representing the 75th percentile of each biomarker across various time points.

	75th percentile MR-proADM (nmol/L)	***75th percentile CT-proET-1 (pmol/L)***	***75th percentile PCT (ng/mL)***	***75th percentile CRP (mg/dL)***
Day 1	*≥ 1.543*	*≥ 146.600*	*≥ 7.420*	*≥ 28.640*
Day 5	*≥ 1.090*	*≥ 93.485*	*≥ 3.490*	*≥ 13.370*
Day 30	*≥ 0.920*	*≥ 84.338*	*≥ 0.060*	*≥ 0.710*

Supplemental Data Table S2. Logistic univariate regression between biomarkers/PSI score and outcomes across various time point, as expressed in Odds Ratio (95% Confidence Interval). ns=not significant.

	In-hospital CVE	***In-hospital mortality***	***ICU admission***
Day 1			
MR-proADM	4.88 (2.80-8.51); p<0.001	6.17 (2.40-15.85); p<0.001	9.92 (4.29-22.95); p<0.001
CT-proET-1	5.63 (3.22-9.86); p<0.001	7.73 (2.90-20.58); p<0.001	5.60 (2.71-11.56); p<0.001
PSI score	4.20 (2.74-6.45); p<0.001	12.97 (4.57-36.80); p<0.001	3.41 (2.11-5.52); p<0.001
PCT	2.20 (0.95-5.09); ns	0.89 (0.23-3.36) ns	5.30 (2.60-10.78); p<0.001
CRP	1.09 (0.70-1.69); ns	1.45 (0.72-2.93) ns	3.14 (1.99-4.90); p<0.001
Day 5			
MR-proADM	5.15 (2.54-10.44); p<0.001	17.21 (3.68-80.67); p<0.001	
CT-proET-1	5.26 (2.59-10.68); p<0.001	6.69 (1.95-22.97); p<0.001	
PCT	2.21 (0.73-6.65); ns	2.31 (0.59-9.15); ns	
CRP	2.10 (1.18-3.72); p=0.012	1.97 (0.84-4.66); ns	
	CVE at 1-year follow-up	***Mortality at 1-year follow-up***	
Day 30			
MR-proADM	6.74 (3.01-15.07); p<0.001	1.82 (0.42-7.81); ns	
CT-proET-1	4.97 (2.26-10.91); p<0.001	1.85 (0.43-7.94); ns	
PCT	1.89 (0.88-4.07); ns	1.21 (0.28-3.17); ns	
CRP	1.79 (0.78-4.12); ns	1.50 (0.27-8.39); ns	

Supplemental Data Table S3. Accuracy of biomarkers/PSI score and outcomes across various time measurements, as expressed in Area Under Curve (AUC). ns=not significant.

	In-hospital CVE	***In-hospital mortality***	***ICU admission***
Day 1			
MR-proADM	0.75 (0.68-0.81); p<0.001	0.86 (0.80-0.91); p<0.001	0.81 (0.72-0.89); p<0.001
CT-proET-1	0.75 (0.69-0.81); p<0.001	0.76 (0.64-0.88); p<0.001	0.75 (0.67-0.83); p<0.001
PSI score	0.77 (0.73-0.81); p<0.001	0.84 (0.77-0.91); p<0.001	0.72 (0.66-0.78); p<0.001
PCT	0.53 (0.40-0.66); ns	0.57 (0.38-0.76); ns	0.72 (0.64-0.81); p<0.001
CRP	0.52 (0.46-0.58); ns	0.56 (0.46-0.66); ns	0.67 (0.60-0.73); p<0.001
Day 5			
MR-proADM	0.78 (0.72-0.85); p<0.001	0.84 (0.75-0.93); p<0.001	
CT-proET-1	0.76 (0.68-0.83); p<0.001	0.77 (0.65-0.90); p<0.001	
PCT	0.66 (0.55-0.82); p= 0.014	0.75 (0.59-0.91); p=0.010	
CRP	0.58 (0.50.0.67); p=0.038	0.57 (0.45-0.69); ns	
	CVE at 1-year follow-up	***Mortality at 1-year follow-up***	
Day 30			
MR-proADM	0.79 (0.72-0.87); p<0.001	0.60 (0.39-0.81); ns	
CT-proET-1	0.77 (0.68-0.86); p<0.001	0.63 (0.46-0.80); ns	
PCT	0.60 (0.49-0.70); ns	0.58 (0.35-0.80); ns	
CRP	0.56 (0.45-0.68); ns	0.69 (0.49-0.89); ns	
